# Supporting meaningful participation of older people in core outcome set development

**DOI:** 10.1111/jgs.19179

**Published:** 2024-09-06

**Authors:** Jacqueline Martin‐Kerry, Sion Scott, Jo Taylor, David Wright, Martyn Patel, Jennie Griffiths, Victoria L. Keevil, Miles D. Witham, Allan Clark, Ian Kellar, David Turner, Debi Bhattacharya

**Affiliations:** ^1^ School of Healthcare University of Leicester Leicester UK; ^2^ Department of Health Sciences University of York York UK; ^3^ School of Pharmacy University of Bergen Bergen Norway; ^4^ Norfolk and Norwich University Hospital Norwich UK; ^5^ Norwich Medical School University of East Anglia Norwich UK; ^6^ Cambridge University Hospitals NHS Foundation Trust Cambridge UK; ^7^ Dept of Medicine, School of Clinical Medicine University of Cambridge Cambridge UK; ^8^ NIHR Newcastle Biomedical Research Centre Newcastle upon Tyne Hospitals NHS Foundation Trust, Cumbria, Northumberland, Tyne and Wear NHS Foundation Trust Newcastle upon Tyne UK; ^9^ Newcastle University Newcastle upon Tyne UK; ^10^ Department of Psychology University of Sheffield Sheffield UK

## INTRODUCTION

The use of core outcome sets (COS) by trials is widely accepted as best practice, aiming to improve research efficiency by enabling comparison and aggregation of results across trials for specific clinical areas.^1^ A COS is an agreed minimum set of standardized outcomes that should be reported in all trials for a specific clinical area.^1^ A COS should include only fundamental outcomes, that is, *core* to evaluating a treatment or intervention, rather than every relevant or important outcome.^1^, ^2^ Trials can additionally measure other outcomes.^1^, ^3^

Outcomes in a COS should be valid and important for all stakeholders. When developing a COS for hospital deprescribing trials,^4^ we involved stakeholders that would be affected by the intervention: older patients and their carers; healthcare professionals who care for older people in hospital; hospital managers; and academics researching older people's medicine/deprescribing. We followed COMET (Core Outcome Measures in Effectiveness Trials) guidance for COS development^1^; this summarizes available methods for COS development but provides limited guidance on how to ensure meaningful involvement of patients, who historically have not been involved in deciding which outcomes should be measured in trials. The INCLUDE framework highlights that older people are often explicitly or implicitly excluded from healthcare research.^5^ Despite anticipating some barriers to older people's participation in our COS study and addressing these in the study planning, we experienced several challenges to ensuring that the selection of outcomes for the COS included their views. We reflect on these challenges, discuss what worked to address them, and present further refinements that could better support equitable, meaningful participation of older people in COS development. **Study registration:** COMET (Core Outcome Measures in Effectiveness Trials) database (https://www.comet-initiative.org/Studies/Details/1825).

## RATING A LONG LIST OF OUTCOMES WAS BURDENSOME FOR PARTICIPANTS

Developing a COS generally begins with generating a long list (10–100 s) of potentially relevant outcomes for participants to rate.^1^ Although a range of techniques exist for approaching consensus, Delphi surveys dominate the literature regardless of the target population and clinical area.^1^, ^6^, ^7^ Guidance states that during the Delphi, participants should consider each outcome individually, without comparing with other outcomes. We presented 49 outcomes (with plain English definitions) in the first Delphi round for participants to rate in terms of their importance to measure in hospital deprescribing trials. We grouped similar outcomes together and provided instructions informed by our Patient and Public Involvement (PPI) members, about how to review each outcome. Despite these strategies, some older people and their carers found it burdensome to rate so many outcomes independently. Some struggled to understand the nature of the intervention and what it was trying to change, and therefore why they were rating outcomes. We believe this may be more pronounced with interventions such as deprescribing that are perhaps not as tangible as a new treatment or device. Based on this experience, we suggest undertaking a multistep process to reduce the number of outcomes presented during the Delphi stage. This could include multi‐stakeholder workshops with smaller numbers of participants earlier to review outcomes ahead of the Delphi and using ranking exercises to support participants to compare and visually rank outcomes.

## OFFERING OLDER PEOPLE DIFFERENT WAYS OF PARTICIPATING

Most Delphi surveys within COS studies are delivered online.^1^ However, to support older people, we provided alternative ways for them to participate: the option to complete the Delphi on paper or by telephone call with a researcher. Most older people preferred to complete a paper copy followed by a telephone call. We found that telephone participation with older people and their carers reduced participant burden by permitting us to explain the purpose of the COS and to support participants through the process. However, some older people may have difficulty hearing. Another mechanism to involve older people meaningfully is face‐to‐face Delphi surveys (we were unable to offer this due to COVID‐19 restrictions). The opportunity for older people to participate in familiar settings such as at home, or in local community venues, can also support their involvement.^5^

## RATING SCALES AND THE DELPHI DID NOT IDENTIFY WHAT WAS “CORE”

A range of rating options can be used when asking COS participants to rate outcomes in a Delphi survey, including a simple “yes/no” (for importance) and Likert scales (e.g., 3, 5, 7, and 9 point). There is no guidance on which to use, although Delphi software developed by the COMET team, which we used in our study, incorporates the Grading of Recommendations, Assessment, Development and Evaluations (GRADE) scale. The GRADE scale is from 1 to 9, presented in categories: 1–3 “not important,” 4–6 “important but not critical,” and 7–9 “very important or critical.”^8^ However, when developing a COS, we are looking to identify *core* outcomes, not just important ones. Our experience was that participants found it difficult to use the nine‐point scale and expressed confusion about how, for example, a score of 7, 8, or 9 was different when all of these were labeled as “very important or critical.” Delphi participants regardless of participant group, rarely rated outcomes as ‘not important’ (1–3), resulting in no outcomes being excluded. This increased participant burden as the number of outcomes did not reduce through Delphi rounds. Burden was further increased by new outcomes suggested by participants, a process often employed in COS studies.^1^

The Delphi process within COS development rarely invites participants to provide a rationale for their rating of an outcome.^1^ We found that without this information, participants in Round 2 seldom changed their rating from Round 1, as they were unclear why others had chosen a different rating. When they did change a rating, it was usually still within the same category (e.g., changing a 5 to a 6). Our participants did not rate any of our 49 outcomes in Round 1 as “not important”; thus, all 49 progressed to Round 2 for re‐rating. Other COS studies have reported similar findings; for example, a recent study presented 88 outcomes in Round 1 and 85 of these progressed to Round 2, plus 13 new outcomes suggested during Round 1.^9^ Another COS study similarly reported limited change in scores between rounds, referring to “stability of stakeholder opinions.”^10^ We believe it is essential to include the reasons within feedback to facilitate building consensus.

Ranking exercises, that encourage participants to determine which outcomes are the most important relative to other outcomes, are proposed as an alternative to the traditional Delphi process. For example, a “worst‐best rating system” was used to develop a COS for chronic kidney diseases.^11^ Card sorting offers another approach that has been successfully undertaken with different stakeholders,^12^ and The James Lind Alliance Priority Setting partnership^13^ use a single Delphi round followed by ranking activities to reduce the number of priorities considered for inclusion.

## WORKSHOPS AND DISCUSSION ARE VALUABLE FOR COS DEVELOPMENT

Due to the large number of outcomes remaining after the Delphi, we held two workshops. The first workshop was to determine which outcomes from the final Delphi round, based on importance, should be included in the COS. The second workshop was to review the outcomes in terms of feasibility and acceptability of outcome data collection. Our approach was atypical: many COS studies focus on “what” to measure and after finalizing the COS, they consider “how” to measure,^1^, ^14^ often without patient involvement.^15^ Representatives from all stakeholder groups actively participated in both workshops, leading to useful discussions about why outcomes were important, with different perspectives on the reality of data collection. Our experience was that when people had the opportunity to discuss outcomes with others, they could fully consider its importance for evaluating the intervention. We suggest that COS development would benefit from a series of workshops from the outset, enabling stakeholders to understand the study and discuss which outcomes are most important to consider for inclusion in a COS, followed by Delphi surveys with a larger number of participants and fewer outcomes. We acknowledge that this approach would be associated with higher resources and costs; however, the benefits of meaningful inclusion would enable clearer consideration of outcomes.

**FIGURE 1:**
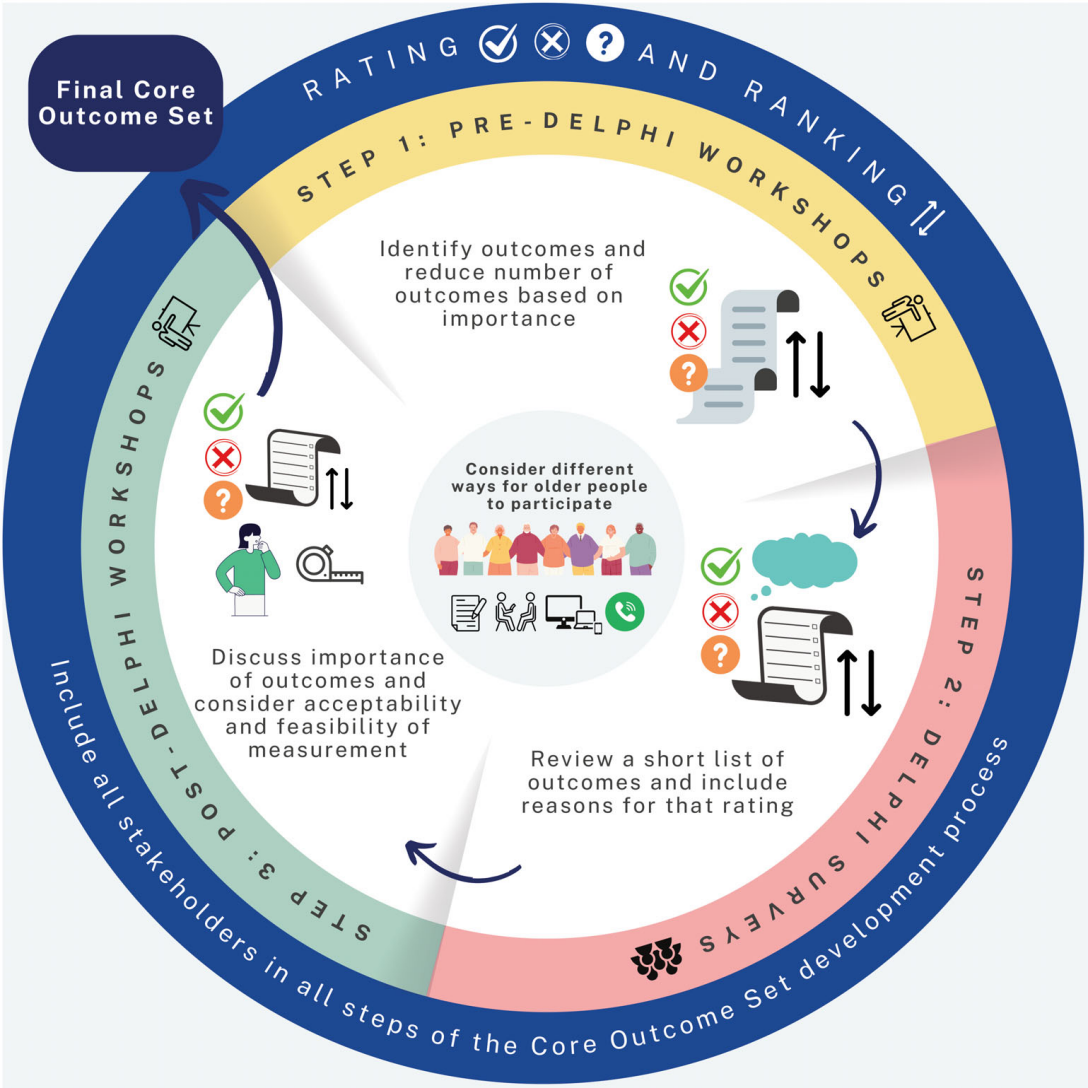
Suggested key steps and considerations when developing a core outcome set (COS) with older people.

Table 1 summarizes our recommendations for future COS development with older people and Figure 1 illustrates suggested key steps and considerations when developing COS with older people.

**TABLE 1:** Key recommendations for future COS development with older people.

Tailor study information to support meaningful participation, recognizing that involvement in the development of a core outcome set will be a new experience for most members of the public and clinicians.
2 Provide a range of options for older people to participate in the process (i.e., face to face, telephone, and online) and when face to face consider familiar settings such as at local community services.
3 Conduct small pre‐Delphi workshops so all stakeholders can consider and discuss potential outcomes in terms of importance.
4 Consider the inclusion of ranking exercises within pre‐Delphi workshops to select relevant and important outcomes to present during the Delphi.
5 Include a smaller number of outcomes in the Delphi process and ensure participants from all stakeholder groups are involved.
6 Consider using an alternative to numerical scales within the Delphi – consider the options ‘yes’, ‘no’, and ‘unsure’ for rating importance.
7 Incorporate the functionality for participants to add a reason for their rating in the Delphi to allow participants in future rounds to consider this information when re‐rating outcomes.
8 Consider ranking exercises within the Delphi activities to support participants to select the most important outcomes.
9 Consider the inclusion of ranking exercises within the post‐Delphi workshops when discussing the importance of outcomes, ahead of consideration of outcome measurement.
10 Ensure that older people are involved in discussions and decisions about the measurement of outcomes in a Core Outcome Set to ensure feasibility and acceptability of data collection.

## CONCLUSION

A valid and reliable COS is important so that all relevant trials are measuring outcomes that are considered *core* by all stakeholders. Developing a valid COS requires meaningful participation and input from those that will be affected. The challenges we experienced are not unique to our study or context.^2^, ^16^, ^17^ Older people however may have certain requirements that need addressing to support their involvement.^5^ We have outlined the methodological approaches we undertook to support their involvement, and identified further refinements that may support meaningful involvement of older patients in COS studies and other research studies. These refinements are likely to be relevant to involving other patient groups. Methodological research is needed to evaluate how these refinements contribute to more meaningful involvement and whether the additional resources required to incorporate additional steps in COS development are worthwhile.

## AUTHOR CONTRIBUTIONS

Debi Bhattacharya, David Wright, Sion Scott, Jo Taylor, Martyn Patel, Ian Kellar, Victoria Keevil, Miles Witham, Allan Clark, and David Turner obtained research funding for the study. Jacqueline Martin‐Kerry conceptualized the idea for the manuscript and wrote the first draft. All authors reviewed and refined the manuscript.

## CONFLICT OF INTEREST STATEMENT

The authors declare no conflicts of interest.

## SPONSOR'S ROLE

The authors declare no role for any sponsor in the preparation of this manuscript.

## FINANCIAL DISCLOSURE

This research study was funded by the National Institute for Health and Care Research (NIHR) Programme Grants for Applied Research stream (award ID PGfAR 200874). This study was supported by the National Institute for Health and Care Research (NIHR) Applied Research Collaboration East of England (NIHR ARC EoE) at Cambridge and Peterborough NHS Foundation Trust. The views expressed are those of the authors and not necessarily those of the NIHR or the Department of Health and Social Care.

## ETHICS STATEMENT

This study was approved by the South Central‐Berkshire Research Ethics Committee (REC Reference number 20/SC/0375) and the UK Health Research Authority (IRAS ID 288286).
